# Assessing the Feasibility of Economic Approaches to Prevent Substance Abuse Among Adolescents: Protocol for a Mixed Methods Study

**DOI:** 10.2196/46486

**Published:** 2023-06-14

**Authors:** Rachel Brathwaite, Massy Mutumba, Jacqueline Nanteza, Lindsey M Filiatreau, Herbert Migadde, Phionah Namatovu, Betina Nabisere, James Mugisha, Abel Mwebembezi, Fred M Ssewamala

**Affiliations:** 1 Brown School Washington University in St. Louis St Louis, MO United States; 2 Department of Health Behavior & Biological Sciences, School of Nursing University of Michigan Ann Arbor, MI United States; 3 International Center for Child Health and Development Masaka Uganda; 4 Department of Psychiatry, School of Medicine Washington University in St. Louis St. Louis, MO United States; 5 Kyambogo University Kampala Uganda; 6 Reach the Youth Uganda Kampala Uganda

**Keywords:** adolescents living with HIV, alcohol, Sub-Saharan Africa, substance use, Uganda

## Abstract

**Background:**

Adolescent alcohol and drug use (ADU) is a significant public health challenge. Uganda, one of the poorest countries in Sub-Saharan Africa (SSA), has the second-highest rate of per capita alcohol consumption in SSA, and over one-third of Ugandan adolescents have used alcohol in their lifetime (over 50% of them engage in heavy episodic drinking). These estimates further increase in fishing villages, a key HIV-vulnerable population, where ADU is normative. However, few studies have assessed ADU among adolescents and youths living with HIV despite their increased risk for ADU and its impact on engagement in HIV care. Moreover, data on risk and resilience factors for ADU are scarce as only few studies evaluating ADU interventions in SSA have reported positive outcomes. The majority have been implemented in school settings, potentially excluding adolescents in fishing communities with high school dropout rates, and none have targeted risk factors including poverty and mental health, which are rampant among adolescents and youths living with HIV and their families, undermine their coping skills and resources, and have been associated with increased risk for ADU among them.

**Objective:**

We propose a mixed methods study with a sample of 200 adolescents and youths living with HIV (aged 18-24 years) seen at 6 HIV clinics in southwestern Uganda’s fishing communities to (1) examine the prevalence and consequences of ADU and identify the multilevel risk and resilience factors associated with ADU among them and (2) explore the feasibility and short-term effects of an economic empowerment intervention on ADU among them.

**Methods:**

This study comprises four components: (1) focus group discussions (FGDs) with adolescents and youths living with HIV (n=20) and in-depth qualitative interviews with health providers (n=10) from 2 randomly selected clinics; (2) a cross-sectional survey with 200 adolescents and youths living with HIV; (3) a randomized controlled trial with a subgroup of adolescents and youths living with HIV (n=100); and (4) 2 postintervention FGD with adolescents and youths living with HIV (n=10 per group).

**Results:**

Participant recruitment for the first qualitative phase has completed. As of May 4, 2023, ten health providers from 6 clinics have been recruited, provided written consent to participate, and participated in in-depth qualitative interviews. Two FGDs was conducted with 20 adolescents and youths living with HIV from 2 clinics. Data transcription, translation, and analysis of qualitative data has commenced. The cross-sectional survey will commence shortly after and dissemination of the main study findings is targeted for 2024.

**Conclusions:**

Our findings will advance our understanding of ADU among adolescents and youths living with HIV and inform the design of future interventions to address ADU among them.

**Trial Registration:**

ClinicalTrials.gov NCT05597865; https://clinicaltrials.gov/ct2/show/NCT05597865

**International Registered Report Identifier (IRRID):**

PRR1-10.2196/46486

## Introduction

### Overview

Adolescent alcohol and drug use (ADU) is a growing public health concern globally, especially in low-resource settings such as Sub-Saharan Africa (SSA), where the epidemics of ADU and HIV/AIDS are co-occurring. ADU plays a significant role in the epidemiology of HIV among adolescents and young adults in SSA, who accounted for over 40% of new infections globally in 2019 [1]. ADU is associated with higher rates of HIV risk behaviors, such as condomless sex [2] and lower rates of HIV testing [3-5]. Among adolescents and youth living with HIV, ADU is a significant barrier to achieving positive HIV treatment outcomes, including enrollment and retention in HIV care [6], adherence to antiretroviral therapy (ART) [7-9], and viral suppression [8,10]. Among HIV-infected adults, it has been shown that alcohol and drug misuse increase AIDS mortality, even among virally suppressed and medication-adherent persons [11-13]. Uganda, one of the poorest countries in SSA, has high rates of HIV/AIDS (6.2%) [14] and alcohol use [15], and illicit drug use is on the increase [16]. Previous studies have reported high rates of alcohol consumption among adults living with HIV [17-19]. Fishing communities, a key vulnerable population in Uganda [20], have high levels of ADU, including among adolescents and youths [16,21-23], which lead to poor HIV prevention and care outcomes.

ADU onset typically occurs in adolescence as an experimental behavior that may escalate into problematic use and disorders [24,25]. Adolescence is a period characterized by the exploration of new roles, identities, and behaviors, including experimentation with ADU [26]. A multitude of factors influence ADU, and these include individual factors (eg, sensation seeking, impulsivity, and mental health) [27-32], interpersonal factors (eg, peer pressure, parental drug use, and poor parental monitoring) [33-43], and structural factors (eg, availability of alcohol and drugs, exposure to ADU marketing, community drug use attitudes, laws and policies, and structural barriers that contribute to poverty) [33,38]. Adolescents and youths living with HIV also experiment with ADU, which may escalate into problematic ADU.

Adolescents and youths living with HIV face numerous HIV-related psychosocial challenges, including HIV stigma, bereavement, chronic pain, relationship stress, and poverty, which heighten their risk for ADU [44-46]. Indeed, research reports indicate a higher burden of mental health problems among adolescents and youths living with HIV [45,47-50], which may lead to ADU. The co-occurrence of mental health problems and ADU is common, including among adolescents and youths living with HIV [49,51-57]. Both ADU and mental health difficulties are associated with nonadherence to ART [48,58-60] and risky sexual behaviors among adolescents and youths living with HIV [59,61-63], which could lead to secondary transmission of HIV and ART resistance due to nonadherence [64-66]. Poor mental health and poverty, which are rampant in poor countries, are significant risk factors for ADU. Poor mental health [54,67,68] and poverty [69-71] are rampant among HIV-affected households, and both are significant risk factors for acquiring HIV [72] and for poor HIV treatment outcomes [73,74]. Adolescents and youths living with HIV living in poverty-stricken households face greater challenges in accessing and sustaining HIV treatment due to economic factors, such as lack of money preventing access to transportation to clinics [75,76] and insufficient resources to afford food leading to inadequate meals to support medication adherence [77-79], which could lead to psychological distress and consequently, ADU [80]. Poverty adversely affects the quality of family relationships, including parent-child communication, involvement [81-83], and parenting skills [84,85], which increases susceptibility to emotional and behavioral challenges and increases risk for ADU [41,43,82,86-90].

Several studies have examined the risk and resilience factors for ADU [91] but few interventions targeting ADU have been tested in SSA. Efforts to prevent ADU are rooted predominantly in the substance use risk reduction and protection enhancement model [91] and require understanding of the risk and resilience factors for ADU. These interventions largely target individual and interpersonal risk factors for ADU, with a focus on providing participants with information on ADU and its consequences or building life skills. In SSA, only 1 intervention targeted the family [92], an important developmental context for adolescents, with a focus on enhancing parenting skills. Yet, none of these interventions has targeted risk factors, such as poverty and mental health, that are widespread in SSA and may undermine the resources and coping skills of adolescents and youths living with HIV.

Despite our knowledge of the higher risk for ADU among adolescents and youths living with HIV [93-96], there is lack of evidence-based interventions targeting ADU risk among adolescents and youths living with HIV. Only 10 ADU interventions have been evaluated in SSA [27,92,97-104] and only a few have been successful [27,98,99,101,103,104]. Most have been ineffective in preventing or reducing ADU, and none of these interventions have targeted adolescents and youths living with HIV. The majority of these interventions are largely school-based [27,100-104], which may exclude adolescents in fishing communities that have high rates of school dropout. These interventions also focused on individual and intrapersonal risk factors for ADU without paying attention to structural risk and resilience factors for ADU. The few existing studies on ADU among adolescents and youths living with HIV have been conducted in high-income countries. These studies focused on younger adolescents, yet ADU typically emerges and escalates in middle-to-late adolescence. Further, these studies rely on self-reports of ADU, which can be undermined by underreporting of ADU.

Economic empowerment (EE) interventions have the potential to prevent ADU among adolescents and youths living with HIV by reducing poverty and its associated mental impacts and bolster adolescents and youths living with HIV and their families’ resources to overcome the challenges associated with HIV. In our previous studies, we have used EE strategies to reduce poverty and improve mental health and HIV care outcomes (eg, medication adherence) among adolescents and youths living with HIV and other AIDS-affected adolescents in Uganda [73,82,105-108]. In this application, we propose to build on this growing evidence by examining the feasibility of using EE to address ADU among adolescents and youths living with HIV.

This study will investigate the epidemiology, underlying risk, and resilience factors for ADU among adolescents and youths living with HIV and evaluate the effects of an EE intervention on ADU among adolescents and youths living with HIV. The specific aims of the study are as follows.

**Aim 1a**: Examine the prevalence and consequences of ADU in a cohort of 200 adolescents and youths living with HIV (aged 18-24 years) seen at 6 HIV clinics in southwestern Uganda. We will use adolescent self-reports and biological measures of ADU (urine).

**Aim 1b**: Using a mixed methods approach, identify the multilevel (individual, interpersonal, community, and structural) factors associated with ADU among adolescents and youths living with HIV.

**Aim 2**: Using a subset of the sample, explore the feasibility and short-term effects of an EE intervention on ADU among adolescents and youths living with HIV.

### Theoretical Framework

**Figure 1:**
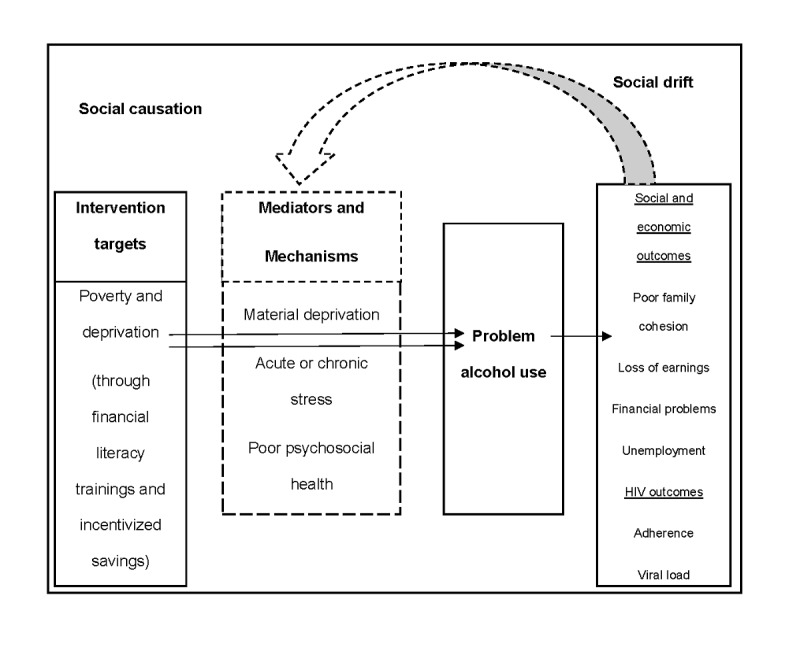
Conceptual model.

Our conceptual approach is informed by the socioecological model (SEM) [109], social causation and drift theories [110], and asset theory [111,112]. We have applied the SEM as a basis for investigating the contextually relevant risk and resilience factors for ADU. SEM posits that environmental factors fall into 4 broad domains: micro-, meso-, exo-, and macrosystems, and interactions within and between these domains determine behavior. This model has demonstrated effectiveness in identifying risk and resilience factors for prevention planning and intervention for ADU [113-117]. Social causation and social drift theories suggest that alcohol consumption problem may be both a response to and a driver of poverty [118]. Acute and chronic stress associated with living in a poverty-impacted environment increases the likelihood of ADU, which causes further material and economic deprivation, hence fueling the cycle of alcohol misuse, and contributing to downward social mobility. Our conceptual framework describes the hypothesized relationship among the intervention targets, poverty, poor mental health, and alcohol use. This has been adapted from Jones and Sumnall [119] (Figure 1)**.** Research in Uganda has reported a higher burden of alcohol and drug abuse among poor populations [120], and many poverty-impacted Ugandan households engage in informal alcohol production for income-generating purposes [121]. Impoverished youths are burdened with hopelessness due to lack of opportunities for improvement in their economic well-being [122,123]. As a result, for impoverished adolescents and youths living with HIV, they may be inclined to spend on instant pleasures, such as alcohol and drugs, as a coping mechanism since they are less likely to believe they can afford the costs associated with accessing and maintaining long-term care for HIV. Our proposed EE intervention is based on Asset theory [124] and is intended to improve economic well-being, relieve poverty and its related consequences, such as poor mental health [106,125], and create a more hopeful and optimistic outlook for the future, thus reducing engagement in risk-taking behaviors like ADU by adolescents and youths living with HIV. The proposed EE will be one of the first studies to examine the impact of EE interventions on reducing ADU among adolescents and youths living with HIV in poverty-impacted communities.

## Methods

### Overall Study Design

**Figure 2:**
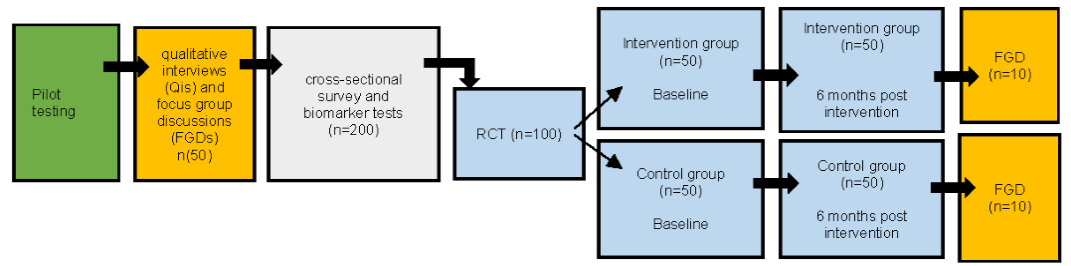
Study components and participant flow through the study. RCT: randomized controlled trial.

This mixed methods study comprises the following four components: (1) focus group discussions (FGDs) with adolescents and youths living with HIV (n=20) and in-depth qualitative interviews (QIs) with health providers (n=10) from 2 randomly selected clinics; (2) a cross-sectional survey (CS) with 200 adolescents and youths living with HIV; (3) a randomized controlled trial (RCT) with a subgroup of adolescents and youths living with HIV (n=100); and (4) 2 postintervention FGDs with adolescents and youths living with HIV (n=10 per study group). Figure 2 depicts the study components and expected number of participants in each component.

### Study Sites and Participants

The study will be conducted in 6 randomly selected HIV clinics located in fishing districts within the Greater Masaka region of Southwestern Uganda—a region heavily affected by HIV (12% prevalence vs 6.2% national average) [126] with high rates of household poverty and alcohol use [127,128]. The target populations for this study are adolescents and youths living with HIV and health care providers at the selected HIV clinics. We will specifically target HIV clinics located in fishing districts with known HIV hot spots that are accredited by the Uganda Ministry of Health as a provider of ART and currently have established services for adolescents and youths living with HIV.

### Inclusion Criteria

For the overall study, the inclusion criteria for adolescents and youths living with HIV are: (1) male or female adolescents and youths living with HIV aged 18-24 years; (2) medically diagnosed with HIV and aware of their HIV status; and (3) enrolled in care at one of the selected HIV clinics. Additionally, we will recruit 1-2 health care providers (psychosocial counselors, nurses, clinical officers, or doctors) from each of the selected clinics. For the RCT, a subsample of adolescents and youths living with HIV (n=100) with a positive self-report for ADU will be randomly selected and randomly assigned at the clinic level (3 clinics per group) to either the intervention group (n=50) or control group (n=50). A subsample of adolescents and youths living with HIV from both the control (n=10) and intervention groups (n=10) will be selected to participate in 2 FGDs at the end of the intervention. Overall, we will exclude any potential participant with significant cognitive impairments that interfere with their understanding of the informed consent process or who is unable or unwilling to consent. For the RCT and postintervention FGDs, we will exclude any adolescents and youths living with HIV with a negative self-report for ADU. However, given the minimum and maximum detection times for different drugs of abuse in the urine, we will not exclude those with a positive self-report but negative urine test results from being included in the RCT.

### Participant Recruitment and Consent

Recruitment strategies will build on Reach the Youth Uganda (local implementing partner) and ICHAD’s (International Center for Child Health and Development) long-standing relationships (>15 years) with 39 health clinics in the greater Masaka region. We will capitalize on recruitment procedures tested in previous SUUBI (Luganda word for Hope) studies that involve collaborating with health clinics in the region (namely Suubi+Adherence: 1R01HD074949-01, Suubi+Adherence-R2: R01HD074949-07, and Suubi4Stigma: R21MH121141) [129-131]. All participants will be recruited from the selected HIV clinics. For the CS, 200 adolescents and youths living with HIV will be recruited from across 6 clinics located in the greater Masaka region. The designated study contact at each health clinic will present the project to all eligible adolescents and youths living with HIV aged 18-24 years during their clinic visits. If there is interest, they will provide verbal consent to be contacted by the research study team. The study coordinator will contact interested adolescents and youths living with HIV to inform them about the required extent of participation, the risks and benefits of participating, and to ask any questions. Written informed consent will be obtained from adolescents and youths living with HIV aged 18-24 years and from health care providers aged more than 18 years to participate. The written consent documents will emphasize the following elements: (1) participation in the study is voluntary; (2) responses to study questions are confidential; (3) participants can terminate their participation at any time, and their decision to withdraw from the study will not affect their access to services they are currently receiving in any way; and (4) participants may be contacted to participate in the RCT if eligible. A screening tool will be developed to assess whether participants meet the study inclusion criteria.

### Ethics Approval

We have obtained approval for the study procedures from the institutional ethics and institutional review boards at the Washington University in St. Louis (202301145) on January 31, 2023 (with an amendment approved on March 2, 2023); the University of Michigan (HUM00223732) on February 23, 2023 and from the in-country local institutional review boards in Uganda: Uganda Virus Research Institute (GC/127/933) on January 16, 2023, and Uganda National Council of Science and Technology (HS2683ES) on March 2, 2023. The study has been registered with ClinicalTrials.gov (NCT05597865) as of October 28, 2022. The dissemination of the main study findings is targeted for 2024. Participants’ data will be protected by a certificate of confidentiality, which protects the privacy of research subjects by prohibiting the disclosure of identifiable, sensitive research information to individuals not involved in the research unless the participant consents.

### Data Collection and Assessments

After pilot testing of data collection interview tools, 2 separate FGDs will be conducted with adolescents and youths living with HIV (n=20) and in-depth QIs will be conducted with health providers (n=10). Adolescents and youths living with HIV will be recruited from 2 randomly selected clinics, with 10 participants per FGD. The FGDs and QIs will explore participants’ perceptions of the multilevel risk and resilience factors associated with ADU and recommendations for culturally appropriate ADU interventions for adolescents and youths living with HIV. Data from the qualitative phase will inform assessments to be conducted in the CS. For the CS, 200 adolescents and youths living with HIV will complete an interviewer-administered survey comprising questions assessing their alcohol and drug consumption patterns and frequency, as well as exposure to multilevel (individual, interpersonal, community, and societal) factors that may be associated with risky and hazardous drinking and drug use among adolescents and youths living with HIV. To reduce social desirability bias, questions on ADU will be self-administered using audio computer–assisted interviewing. We refer to the social ecological framework [132] to guide data collection on the multiple levels of influence that are associated with harmful alcohol and drug use among adolescents and youths living with HIV (see Table 1 for socioecological framework and additional measures that will be collected at each time point). Questions assessing the presence of common mental disorders and other physical health conditions will be included.

Urine tests for ADU will be conducted for all 200 adolescents and youths living with HIV who participate in the CS and for the 100 who participate in the RCT (at the 2 time points: baseline and postintervention). A trained research assistant will collect and test a urine specimen from each participant. The urine sample will be tested for up to 16 classes of the most commonly abused illicit drugs using the T-Cup 16 panel Compact Instant Drug Test Cup at the study site. The T-Cup can detect alcohol in the urine from as early as 8 hours to up to 80 hours after consumption. Minimum and maximum detection times for illicit drugs of abuse range from 1 hour to 40 days, depending on the drug.

**Table 1:** List of measures and time points collected during the study.

Category	Measure	Time point
Individual level of socioecological framework	Sociodemographic: gender, age, orphanhood status, education, income and unemployment, food insecurity [133], experienced homelessness, refugee, day and boarding school, and urban and rural residence. Mental health: depression [134], hopelessness [135], optimism [136], stress [137], pain [138], and history of substance abuse [139].	Cross-sectional survey, qualitative interview, focus group discussion, randomized controlled trial
Interpersonal and relationship levels of socioecological framework	Loneliness [140], number of close friends, bullying, interpersonal violence [141,142], social support [143], family cohesion [144,145], childhood abuse and trauma [146], family history of substance abuse [139], and sexual risk-taking [147]	Cross-sectional survey, qualitative interview, focus group discussion, randomized controlled trial
Community level of socioecological framework	Community norms and access to alcohol and illegal drugs	Cross-sectional survey, qualitative interview, focus group discussion, randomized controlled trial
Society level of socioecological framework	Educational campaigns, price and supply of alcohol, and social stigma [148]	Cross-sectional survey, qualitative interview, focus group discussion, randomized controlled trial
Biomarkers	Urine alcohol and drug tests	Cross-sectional survey, randomized controlled trial
Savings deposits	Savings	Randomized controlled trial
Assessments	Self-reported alcohol and drug use [139], viral load from clinic records, self-reported adherence [149], depressive symptoms [134], hopelessness [135], physical health conditions, and sexual risk-taking [147]	Randomized controlled trial

### Randomized Controlled Trial: Intervention Conditions

For the RCT, 100 adolescents and youths living with HIV with a positive self-report or urine ADU test (based on data from the CS) will be randomly recruited from the 6 clinics and randomly assigned at the clinic level (3 clinics per group) to either the control (n=50) or intervention (n=50) group. The intervention will be delivered over a period of 6 months, with assessments at baseline and 6 months (end of intervention). Upon completion of the RCT, adolescents and youths living with HIV will be randomly selected to participate in 2 FGDs (n=10 from control group and n=10 from intervention group). Adolescents and youths living with HIV in the intervention group will share how the intervention affected their alcohol and drug use as well as their recommendations on how to improve the intervention to be more culturally appropriate and effective in reducing ADU among adolescents and youths living with HIV in Uganda, while adolescents and youths living with HIV in the control group will share their experiences with ADU risk reduction sessions and how this influenced their ADU.

### Description of the Control Condition

Adolescents and youths living with HIV in the control condition will receive 4 ADU risk reduction sessions tailored for adolescents and youths living with HIV. Working with author JM (consultant), we plan to adapt, expand, and tailor the Program for Appropriate Technology in Health (PATH) Life Planning Skills curriculum (Unit 11 on substance use) [150] to include issues specific to adolescents and youths living with HIV. We will train research assistants, using an adapted facilitator’s manual, to deliver the adapted curriculum. As part of the RCT, adolescents and youths living with HIV will complete a questionnaire (at baseline and at the end of the intervention) on their alcohol and drug use behaviors (self-reported and biologically measured) and risk and resilience behaviors.

### Description of the Intervention Group

In addition to the adapted ADU training module that the control group will receive, adolescents and youths living with HIV in the intervention arm will receive 4 financial literacy training sessions and a youth development savings account (YDA) for long-term savings. Although akin to conditional cash transfer interventions, which have become increasingly popular in the social development field by enabling individuals to meet basic needs while incentivizing prosocial behaviors [82,107,151-153], EE interventions that apply matched savings accounts go beyond incentivizing behavior. They emphasize long-term investment and promote lifelong financial inclusion by forming savings habits and establishing partnerships between the participants and local financial institutions and the actual intervention program. For the proposed study, the EE intervention will be in the form of a YDA, where savings are housed at a local bank and deposits made by the adolescent are matched by the intervention to encourage savings. YDAs yield positive effects, including creating a greater sense of security, self-confidence, and future orientation for young people [82,107,151-153]. Each adolescent and youth living with HIV in the EE intervention will receive a YDA held in their own name in a bank registered by the Central Bank (Bank of Uganda). We will form partnerships with national banks operating in the study area. The account will then be matched with money from the program. The maximum adolescent’s contribution to be matched by the program will be an equivalent of US $20 per month per adolescent or US $120 for the 6-month intervention period. Our previous studies indicate that the partner financial institutions have multiple and easily accessible deposit locations in the study area and that participants can save these amounts [82,107,151-153]. Therefore, adolescents and youths living with HIV who save the maximum amount will have a total of US $240 at the end of the intervention (US $120 in savings and US $120 from the match: a 1:1 match).

As in the studies that inform this application, each month a bank account statement will be generated for every adolescent and youth living with HIV to note their accumulated savings. The statements are intended to act as “moral boosters” for the enrolled adolescents and youths living with HIV. Unique to this study is our innovative spending model, which empowers participants to make informed financial decisions. During the intervention period, adolescents and youths living with HIV will have direct access to both their personal savings deposited in the accounts as well as the match provided by the study. This is different from our previous studies that required the participants’ own savings and the match to be maintained in separate accounts and to get approval by the research team to access the match [82,107,151-153]. This added unconditional component provides adolescents and youths living with HIV with a safety net to address short-term medical needs and financial and consumption emergencies if they arise. Participants will be provided with financial literacy sessions and mentorship tailored specifically to the needs of adolescents and youths living with HIV and ADU. We expect adolescents and youths living with HIV to be equipped with the knowledge to make well-informed consumption and expenditure decisions but also to feel supported in case of immediate medical needs. The research team will monitor, but not restrict, how participants spend their money. Additionally, the study team will have access to and review participants’ bank statements to ascertain deposit and withdrawal frequency. Participants will be encouraged to use financial diaries to record their expenditures.

### Analysis of CS Data

Frequency distributions and summary statistics for the outcome and all predictor variables will be derived. The prevalence of ADU from both biological tests and self-reports, assessed using the Smoking and Substance Involvement Screening Test (NIDA-Modified ASSIST) [139] among adolescents and youths living with HIV, will be calculated by dividing the number who tested positive for ADU by the total number of participants tested and dividing the number screened to be of moderate or high risk in self-reported data by the total number of participants who completed the survey. To determine which multilevel factors are associated with substance misuse, we will fit logistic regression models comprising the outcome (ADU) and potential risk factors with standard errors adjusted for clustering by clinics.

### Analysis of RCT Data

#### Primary Outcome Measures

Our primary outcomes are feasibility and acceptability of the intervention. Feasibility will be determined by the proportion of participants who enrolled or refused to participate in the intervention. In addition, we will measure the willingness of local partners to assist with recruitment, reasons for refusal, enrollment, and ineligibility. Acceptability will be determined by measuring retention rates at 6 months, the proportion of participants that adhered to study procedures, intervention attendance and engagement, and the extent to which the intervention is acceptable and appealing to study participants.

#### Secondary Outcome Measures

Changes in ADU and mental health functioning are our secondary outcomes. Our primary hypothesis is that participants in the intervention group will have a lower odds of ADU compared to participants in the control group at the end of the intervention. Our secondary hypothesis is that adolescents and youths living with HIV in the intervention group will have better mental health outcomes, including lower levels of depressive symptoms, less hopelessness, improved adherence, better viral suppression, and improved economic outcomes than adolescents and youths living with HIV in the control group. To test these hypotheses, we will fit 3-level mixed-effects models. Each model will comprise the outcome and fixed categorical effects for study group (intervention vs control), time (baseline and 6-months), and a group-by-time interaction term. Random intercepts will be fitted at the clinic and person levels, with unstructured correlations among subjects’ repeated measures. Robust Huber-White standard errors and test statistics will be computed for each model. We will assess the omnibus effects of study group, time, and their interaction. Postestimation analyses will be conducted to assess time within group simple effects and group within time simple effects.

### Qualitative Data Analysis

Interviews will be transcribed and uploaded to NVivo 12 (QSR International) [154]. Analytical induction techniques [155] will be used for coding. Ten interview transcripts will be randomly selected, read multiple times, and independently coded by the team using sensitizing concepts to identify emergent themes (open coding) [156]. Broader themes will be broken down into smaller, more specific units until no further subcategory is necessary. Analytic memos will be written to further develop categories, themes, and subthemes and to integrate the ideas that emerge from the data [156,157]. Codes and the inclusion and exclusion criteria for assigning codes will be discussed as a team to create the final codebook in NVivo 12. Each transcript will then be independently coded by 2 investigators using the codebook. Intercoder reliability will be established. A level of agreement ranging from 66% to 97% based on level of coding indicates good reliability [158]. Disagreements will be resolved through team discussions. The secondary analysis will compare and contrast themes and categories within and across groups to identify similarities, differences, and relationships among the findings. Member checking, peer debriefing, and an audit trail will be used to ensure rigor [159].

## Results

Recruitment of participants for the first qualitative phase has commenced. As of May 4, 2023, ten health providers from 6 clinics have been recruited, provided written consent to participate, and participated in in-depth QIs. Two FGDs was conducted with 20 adolescents and youths living with HIV from 2 clinics. Data collection for the qualitative phase has completed and transcription, translation, and analysis of qualitative data will commence immediately thereafter. The CS will begin shortly after the qualitative phase. Data collection will continue over the next year, and dissemination of the main study findings is targeted for 2024.

## Discussion

This will be one of the first studies to examine the impact of EE interventions on reducing alcohol and drug use among adolescents and youths living with HIV in poverty-impacted communities. This study will advance our understanding of the epidemiology, underlying risk factors, and consequences of ADU among adolescents and youths living with HIV in high-risk environments and evaluate a culturally tailored intervention that could prevent harmful ADU and improve the overall and long-term health and well-being of adolescents and youths living with HIV. We specifically target adolescents and youths living with HIV living in fishing communities—known HIV hot spots that typically have high levels of poverty as well as ADU. Study findings will contribute to our understanding of the epidemiology, risk and resilience factors, and consequences of ADU among adolescents and youths living with HIV in order to inform the development of effective ADU prevention interventions. We envision that the findings will also inform the design of longitudinal studies to evaluate the long-term effects of an EE intervention on ADU among adolescents and youths living with HIV.

There is a scarcity of evidence-based interventions for addressing ADU among youth in resource-limited settings. Only a few ADU interventions evaluated in SSA have targeted the family context [92]. No study has targeted poverty and its attendant impacts (eg, mental health) as a risk factor for ADU. In this proposal, we will examine the feasibility, acceptability, and short-term effects of an EE intervention to reduce and prevent risky and hazardous alcohol and drug use. Our intervention targets the most commonly occurring risk factors for ADU (ie, poverty and mental health problems). This will be one of the first studies to examine the impact of EE interventions on reducing alcohol and drug use among adolescents and youths living with HIV in poverty-impacted communities. The proposed EE intervention is based on Asset theory [124] and is intended to improve economic well-being, create a more hopeful and optimistic outlook for the future, and thus reduce engagement in risk-taking behaviors by adolescents and youths living with HIV, including ADU. However, our findings should be interpreted with some limitations. Given our target population, our findings may not be generalizable to adolescents and youths living with HIV populations in more urban or developed areas, and to younger or older age groups. This is a small feasibility study, so our study is not powered to detect significant changes but will provide preliminary data for a larger-scale study with sufficient power.

## Appendix

Multimedia Appendix 1 Summary statement for grant reviewed by Center for Scientific Review Special Emphasis Panel Member Conflict: HIV/AIDS Related Behavioral Research.

## Abbreviations

ADU: alcohol and drug use
ART: antiretroviral therapy
CS: cross-sectional survey
EE: economic empowerment
FGD: focus group discussion
ICHAD: International Center for Child Health and Development
PATH: Program for Appropriate Technology in Health
QI: qualitative interviews
RCT: randomized controlled trial
SEM: socioecological model
SSA: Sub-Saharan Africa
YDA: youth development savings account

## Data Availability

Data sharing is not applicable to this study as no data sets were generated or analyzed during this study. However, the data sets generated during this study will be available in the National Institute of Mental Health Data Archive.
