# Supplementary material for: Assessing the Feasibility of Economic Approaches to Prevent Substance Abuse Among Adolescents: Protocol for a Mixed Methods Study
Source: JMIR Res Protoc. 2023 Jun 14;12:e46486. doi: 10.2196/46486 (PMC10337321; doi:10.2196/46486)
Supplement: Multimedia Appendix 1 [file resprot_v12i1e46486_app1.pdf]

SUMMARY STATEMENT
(Privileged Communication)

PROGRAM CONTACT:
Dr. Robert Freeman
301443-8820
rfreeman@mail.nih.gov

Release Date: **12/13/2021**
Revised Date:

Application Number: **1 R21 AA030225-01**

Principal Investigators (Listed Alphabetically):

BRATHWAITE, RACHEL (Contact)
MUTUMBA, MASSY

Applicant Organization: WASHINGTON UNIVERSITY

Review Group: **ZRG1 RPHB-T (04)**
Center for Scientific Review Special Emphasis Panel
Member Conflict: HIV/AIDS Related Behavioral Research

AIDS

Meeting Date: **12/03/2021**
Council: **JAN 2022**
Requested Start: **04/01/2022**

RFA/PA: **PA20-194**
PCC: **APAF**
Dual PCC: **EB/JAV**
Dual IC(s): **DA, HD**

Project Title: **Assessing the Feasibility of Economic Approaches to Prevention of Substance Abuse among Adolescents**
SRG Action: **Impact Score:38**
Next Steps: **Visit https://grants.nih.gov/grants/next_steps.htm**
Human Subjects: **48-At time of award, restrictions will apply**
Animal Subjects: **10-No live vertebrate animals involved for competing appl.**
Gender: **1A-Both genders, scientifically acceptable**
Minority: **5A-Only foreign subjects, scientifically acceptable**
Age: **1A-Children, Adults, Older Adults, scientifically acceptable**

Project Year	Direct Costs Requested	Estimated Total Cost
1	125,000	191,668
2	150,000	230,002
TOTAL	275,000	421,670

ADMINISTRATIVE BUDGET NOTE: The budget shown is the requested budget and has not been adjusted to reflect any recommendations made by reviewers. If an award is planned, the costs will be calculated by Institute grants management staff based on the recommendations outlined below in the COMMITTEE BUDGET RECOMMENDATIONS section.

1R21AA030225-01 Brathwaite, Rachel

PROTECTION OF HUMAN SUBJECTS UNACCEPTABLE

RESUME AND SUMMARY OF DISCUSSION: This study will examine the impact of a family-based economic empowerment intervention on reducing alcohol and drug use (ADU) among adolescents and youth living with HIV (AYLHIV) in poverty-impacted communities in Uganda.The rigor of the prior research is strong and the high significance of the study lies in the ability to advance what is currently known about high risk AYLHIV for whom few effective AUD interventions exist. This is an outstanding investigative team who are well suited to the project. The study was considered innovative given its focus on a high priority population. Additional strengths of the study noted during the discussion included the detailed sampling and recruitment strategies, the strong dissemination plan, the embedding of data collection with medical visits, the use of RCT outcome measures that are well described and feasible, and the methods for qualitative data collection and analysis that are well described. Some minor and addressable weaknesses were noted around the lack of detail for the rationale for Aim 1; the logistics of the family-based intervention that lacked detail; the underdeveloped discussion of interventionists training; the need for a more thorough literature review of psycho-social interventions; the concern that the study population age range spanned large developmental differences which could limit study feasibility; and the timeline which was considered overly ambitious. Some human subjects' concerns were also noted around data confidentiality and minors. These weaknesses did not affect the overall impact of the study. Following the discussion the proposed project was seen as potentially very important with a high probability of success. Findings are expected to have a high overall impact on the field.

DESCRIPTION (provided by applicant): Adolescent alcohol and drug use (ADU) is a significant public health challenge in sub-Saharan Africa (SSA). About 41.6% of adolescents in SSA reported using at least one psychoactive agent, with alcohol being the most commonly used drug. Uganda, one of the poorest countries in SSA, has the second highest rate of per capita alcohol consumption in SSA (15.1 liters of pure alcohol vs regional average of 6.2 liters of pure alcohol) and over one third of Ugandan adolescents have used alcohol in their lifetime, 22.5 million are current drinkers, and over 50% engage in heavy episodic drinking. These estimates reach even greater magnitudes in the country's fishing villages – a key HIV vulnerable population- where ADU is normative. A few studies have assessed ADU among AYLHIV, yet AYLHIV are at higher risk for ADU, and ADU impedes adherence to anti-retroviral therapy (ART), retention in care, and viral suppression. Several studies have examined the risk and resilience factors for ADU but few interventions targeting ADU have been tested in SSA. Only 10 ADU interventions have been evaluated in SSA and only a few have been successful. The majority have been implemented in school settings, which may exclude adolescents in fishing communities that have high rates of school dropout. Moreover, none has targeted risk factors such as poverty and mental health, which are rampant among AYLHIV and their families, undermine AYLHIV's coping skills and resources, and have been associated with increased risk for ADU among adolescents. Family-based economic empowerment (FEE) interventions have the potential to prevent ADU among AYLHIV by reducing poverty and its associated mental health impacts, and also bolstering AYLHIV and their families' resources to overcome the challenges associated with HIV. Given the lack of evidence-based culturally tailored interventions to prevent ADU in AYLHIV in low-income settings such as Uganda, this study proposes to: Aim 1a. Examine the prevalence and consequences of ADU in a sample of 200 AYLHIV (ages 15- 24) seen at six (6) HIV clinics located in the fishing communities of southwestern Uganda. ADU will be measured using self-report and biological data (i.e. urine). Aim 1b. Using a mixed methods approach, identify the multi- level (individual, interpersonal, community and structural) factors associated with ADU among AYLHIV. Aim 2: Using a subset of the sample, explore the feasibility and

short-term effects of a FEE intervention on ADU among AYLHIV. Our intervention focuses on older adolescents and young adults in a high-risk environment (i.e. fishing communities) to elucidate the contextually relevant risk and resilience factors for ADU among AYLHIV undergoing social transitions. Additionally we innovatively target the most commonly occurring risk factors for ADU (i.e. poverty and mental health problems) through the FEE that includes provision of youth development savings accounts, financial literacy sessions and ADU risk reduction sessions. Our findings will inform the design of an R01 grant to examine the long-term effects of a family-based economic empowerment intervention on ADU among AYLHIV.

PUBLIC HEALTH RELEVANCE: The proposed study will be the first to innovatively examine the impact of family-based economic empowerment intervention on reducing alcohol and drug use (ADU) among adolescents and youth living with HIV (AYLHIV) in poverty-impacted communities in Uganda, and focuses on improving understanding of multi-level context- specific risk and protective factors for ADU among AYLHIV. Given that many ADU interventions evaluated in sub-Saharan Africa have not been successful, study findings could inform interventions to reduce/prevent ADU, and also improve our understanding of the epidemiology, risk and resilience factors and consequences of ADU among AYLHIV, in order to inform the development of effective ADU prevention intervention. Lessons learned from implementing ADU prevention efforts in low-income countries such as Uganda, may be relevant to improving overall care for other vulnerable adolescent populations in sub-Sahara Africa where the burden of ADU is increasing yet strategies to alleviate the burden of ADU among young people at high risk are lacking.

CRITIQUE 1

Significance: 4
Investigator(s): 2
Innovation: 3
Approach: 5
Environment: 1

Overall Impact: The study proposes to investigate the epidemiology, underlying risk and resilience factors for alcohol and drug use (ADU) among older adolescents and young adults living with HIV (AYLHIV) in Uganda, and to evaluate the effects of a family-based economic empowerment intervention on ADU among AYLHIV in the setting (fishing communities in southwestern Uganda) In Aim 1 a cross-sectional study is proposed to examine prevalence and consequences of ADU (self-report and a urine assay) in a sample of 200 AYLHIV (ages 15- 24) seen at six (6) HIV clinics in the setting. Investigators aim to identify "the multilevel (individual, interpersonal, community and structural) factors associated with ADU among AYLHIV". In Aim 2, investigators propose a pilot study to assess explore the feasibility and short-term effects of an economic intervention on ADU among AYLHIV. The intervention consists of four (4) Financial Literacy (FL) training sessions and a Youth Development Savings account (YDA) for long-term savings, which family members can access and which is incentivized with matching funds from the study. Enthusiasm for the study is dampened by several weaknesses in the study premise, study design and approach. First, given prior research in this area, the rationale for the Aim 1 study to explore the multi-level factors that contribute to alcohol and drug use among adolescents is not convincingly explained. Second, evidence in support of the premise of the intervention design was not strong – the intervention is comprised of an adapted life skills training program with the addition of a family savings intervention – there is no component in this intervention of an evidence-based psychological/ mental health or counseling based intervention element. Given that the stated intent is also to improve mental health outcomes, the absence of a clear premise based on

prior psychosocial and therapeutic theory and research is notable. The investigators do not explain why the standard counseling-based approaches in substance abuse intervention research are not included. One would have expected a thorough review of prior interventions in the field that have proven successful in ADU reduction but which may need to be adapted for the population, or may be enhanced if combined with an economic intervention. The rationale for a stand-alone economic approach (with the addition of a standard life skills curriculum, ubiquitous in the setting and the control condition for the study) is not clearly offered. The logistics of the intervention and what makes it family-based not spelled out. It was not clear how the study would ensure adolescents would have control over spending, nor how family members would be involved in training sessions. There is a lack of clarity of the stated measurements for the RCT: the outcome measures are not standard for this area of research and there is inadequate description of statistical analysis plans, with a mismatch between what is described in the research strategy (with a stated outcome of RCT given as ADU) and what is included in the statistical analysis plan (outcomes are measures of acceptability and feasibility of RCT, with no mention of alcohol and drug use). Further, there is inconsistency in language used regarding emphasis on alcohol vs. other drug use, and it is unclear whether or not the intervention is intended to address multiple substances. The MPIs have no prior experience as PIs of an R-series grant, and no track record of collaboration in terms of prior publications together (although both have collaborated with mentor and Co-I Dr. Ssewemala). The timeline for the study appears to be over-ambitious, especially in the activities planned for year 2. The investigators are to be commended for a focus on a high priority population in need of services, and the intent to explore upstream drivers of alcohol and drug use among adolescents and young adults in the setting.

1. Significance:

Strengths

- The study addresses a high priority population and a significant public health problem: Adolescent alcohol and drug use (ADU) is a significant public health challenge in SSA. a key driver of new HIV infections and poor HIV care outcomes in SSA.
- The co-occurrence of mental health problems and ADU is common, including among AYLHIV, and is associated with poor HIV outcomes in the setting.
- Poverty is a significant risk factor for HIV acquisition and poor treatment outcomes and operates through a range of mechanisms. AYLHIV living in poverty-stricken households face greater challenges in accessing and sustaining HIV treatment due to economic factors such as food insecurity and lack of money for transport. Economic interventions may play an important role in sustaining improved health outcomes in high-risk populations.
- Evidence-based culturally tailored interventions to prevent ADU in AYLHIV are few in number in sub-Saharan Africa.

Weaknesses

- The intervention is proposed to address multiple links between poverty and poor mental health and alcohol use outcomes, but the causal chain does not clearly describe hypothesized relationships, and the literature cited includes few references. A diagram of the theoretical framework would have been helpful, to better clarify hypothesized causal relationships and intervention targets.
- Investigators argue that poverty adversely affects the quality of family relationships- which in turns increases risk of ADU in adolescents and young adults. But the intervention itself does not seem to target family relationships, or to be designed to strengthen them. It is not clear how the

quality of family relationships- if important to the hypothesized link between the intervention and the improved outcomes- would be measured in this study.

- Investigators cite a large literature on the “multitude of factors [that] influence ADU” and these include “individual factors (e.g., sensation seeking, impulsivity, mental health),79-84 interpersonal factors (e.g., peer pressure, parental drug use, poor parental monitoring)85-95 and structural factors (e.g., availability of alcohol and drugs, exposure to ADU marketing, community drug use attitudes, laws and policies).” With this knowledge base, is the research under Aim 1 necessary? The gaps in the existing literature and how this study would address them is not convincingly described.
- Team asserts that “for impoverished AYLHIV, they may be inclined to spend on instant pleasures such as alcohol and drugs as a coping mechanism since they are less likely to believe they can afford the costs associated with accessing and maintaining long-term care for HIV” but does not cite an evidence base for this assertion; how would the investigators be confident that increased income from the savings intervention would not lead to more spending on alcohol?

2. Investigator(s):

Strengths

- MPI Dr. Brathwaite has a strong publication record in substance use and HIV given her very early career stage. MPI Mutumba has also published many articles on alcohol and substance use, and adolescents living with HIV in Uganda, and has experience as Co-I on prior studies on this topic in the region.
- Dr. Brathwaite has research experience in substance use disorders and mental health among vulnerable/marginalized populations. She has published research focused on developing tools designed to predict future depression among adolescents in low and middle income countries and to predict depression and hopelessness among adolescents living with HIV in Uganda.
- Dr. Mutumba has been involved in HIV and AIDS related clinical care and research. Her research interests focus on the intersections of mental health, substance use and health outcomes among adolescents.
- Mentor and Co- I Dr. Fred Ssewamala’s is a very senior and experienced researcher with PI status on multiple NIMH, NICHD and NCI randomized clinical trials implementing economic empowerment interventions for families and children affected by HIV and women engaged in sex work.
- Drs. Brathwaite and Ssewemala have published together.
- The research team includes a strong team of Ugandan Co-Investigators, with experience collaborating (Ssewemala, Mugisha, Mwebembezi) on mutliple research projects.

Weaknesses

- The MPI team has limited prior experienced with managing research studies as PI/MPI. PI Brathwaite is a Postdoctoral Research Associate at Washington University (PhD awarded in 2017); MPI Mutumba is Assistant Prof at U of Michigan (PhD 2015). Brathwaite holds a supplement as MPI to an existing D43 project unrelated to this research (pertaining to institutional capacity to address sexual harassment.) She also lists role as Fellow to an existing R25 held by Ssewamala and colleagues. No other / prior support is listed. Dr. Mutumba only lists current intramural grants. No other / prior PI grants listed.

- Mutumba has not yet published with Brathwaite, or Ssewemala, but is currently working on a study with Ssewemala. MPIs do not yet have a track record of collaboration.

3. Innovation:

Strengths

- The focus on alcohol and drug use among older adolescents and young adults in a high-risk environment (fishing communities in Uganda) and on poverty and mental health problems as risk factors for ADU may be innovative.
- The study may be among few studies to examine the impact of economic interventions on reducing alcohol/drug use among AYLHIV in poverty-impacted communities

Weaknesses

- The use of biomarker data to address limitations of self-reported drug use is standard, not an innovation per se.

4. Approach:

Strengths

- The qualitative and mixed methods approach and analysis procedures well-described for the Aim 1 study. The FGDs and QIs will explore participants' perceptions on the multi-level risk and resilience factors associated with alcohol and drug use and recommendations for culturally appropriate ADU interventions for AYLHIV. Data from the qualitative phase will inform the questions to be asked in the cross-sectional survey.
- Sampling and recruitment strategies for the FGDs and interviews appear appropriate.
- The RCT sample design appear appropriate; 100 AYLHIV with a positive self-report or urine ADU test (based on data from the cross-sectional survey) would be "randomly recruited from the six clinics and randomly assigned at the clinic level (3 clinics per group) to either the control (n = 50) or intervention (n = 50) group". The intervention will be delivered over a period of six months, and with assessments at baseline, and 6 months (end intervention).
- The savings matching may be promising for moderately incentivizing savings contributions to the Youth Development Savings account (YDA). (The maximum family contribution to be matched by the program will be an equivalent of US $20 per month per family or US $120 for the 6-month intervention period; this may be appropriate.)

Weaknesses

- Inadequate review of prior alcohol use reduction interventions - The rationale does not include review of tested interventions to reduce alcohol use in the setting,
- There is no explicit behavior change / counseling / psychological component to address psychological and behavioral aspects or ADU reduction; it is unclear why the investigators feel an economic intervention alone will be effective at reducing ADU, and does not explain why other psychosocial, behavioral counseling or other family support elements are not needed.
- Is the intervention designed to prevent ADU, or to help adolescents living with HIV reduce ADU? This is not clear.
- The team plans to "adapt, expand and tailor the Program for Appropriate Technology in Health (PATH) Life Planning Skills curriculum (Unit 11 on substance use) to include issues specific to

AYLHIV". Then, will "train research assistants, using an adapted facilitator's manual, to deliver the adapted curriculum". No description of the adaptation process is given.

- In addition to the adapted ADU training module that the control group will receive, AYLHIV in the intervention arm will receive four (4) Financial Literacy (FL) training sessions and a Youth Development Savings account (YDA) for long-term savings. Why is a life skills curriculum chosen as an element of the intervention? Has it been shown to have any effect on substance use?
- The cross-sectional survey and urine sample collection will be conducted among 200 AYLHIV; the procedures for the assay are not well-described.
- "The research team will monitor, but not restrict, how families spend their match." How would study ensure the adolescent / YA has power to access the money / control spending? How would study ensure the money is spent on target participants and not on other family needs and priorities?
- The inclusion criteria for AYLHIV: 1) male or female AYLHIV aged 15-24 years; - such a wide age span inclusive of adolescents living with parents as well as those who are independent—the definition of 'family' vis a vis the intervention is not clearly explained. How might the intervention implementation and effectiveness differ for younger adolescents living with parents' vs older adolescents and young adults living independently or starting their own families?
- How might the intervention need to be tailored by gender, or how will gender be addressed and analyzed?
- "Any of the AYLHIV's family members, relatives, or friends will be allowed and encouraged to contribute towards the YDA." Can they also withdraw money? How will this be managed, with the younger adolescents' co-resident with parents vs those who are starting their own families?
- For the RCT, the power calculation for the primary hypothesis not shown. Investigators state "We hypothesize that participants in the intervention group will have a lower odds of ADU compared to participants in the control group." In the statistical analysis section, power calculations based on measurements of acceptability and feasibility, not ADU. ("formal hypothesis testing will not be the study focus"), yet measures of feasibility and acceptability not described within the Aim/
- Investigators aim to address a range of outcomes over a short time period ("We will implement the intervention over 6 months (July–December 2023)". Team hypothesizes that "AYLHIV in the intervention group will have better mental health outcomes including lower levels of depressive symptoms, less hopelessness, improved adherence and achieve better viral suppression and improved economic outcomes than AYLHIV in the control group." It may be over ambitious to assume these measures would all appreciably improve over the time period. Procedures for assessing changes in measures are not described.
- Outcome measurements include prevalence of ADU from both biological tests and self-reports, assessed using the Smoking and Substance Involvement Screening Test (NIDA-Modified ASSIST) among AYLHIV, and the urine sample will be tested for up to 16 classes of the most commonly abused illicit drugs using the T-Cup 16 panel Compact Instant Drug Test Cup at the study site. "The T-Cup can detect alcohol in the urine from as early as 8 hours to up to 80 hours after consumption". The rationale and appropriateness of use of these measures and assay in this setting and population is not clearly explained.
- Biological data will include urine for ADU tests and HIV viral load to be abstracted from patient medical records. HIV viral load data are collected every six months as part of the HIV care

monitoring. How will quality of the VL data be assessed? How can study team ensure that VL data temporally aligned with measurement periods for the study (baseline, follow up) given that schedules for VL data collection highly variable in routine clinical care environments? What about for study participants who are not currently in care or receiving regularly VL assessments?

5. Environment:

Strengths

- The environment at Washington University, University of Michigan, and the International Center for Child Health and Development (ICHAD) in Uganda (founded by Co-I Dr. Ssewemala) are all excellent for the research.
- Currently ICHAD houses several longitudinal NIH funded field studies that examine the efficacy and cost effectiveness of economic empowerment interventions for poor youth and families across sub-Saharan Africa; and several NIH funded training grants and supplements (R25, T37 and D43).
- Reach the Youth (RTY) is an implementing NGO partner, and works with AIDS-affected children and adolescents in districts of southwestern Uganda; Co-I Mwebembezi is executive director.

Weaknesses

- None noted.

Study Timeline:

Strengths

- The timeline of activities in the first year appears appropriate.

Weaknesses

- Timeline in year two may not be feasible. As shown, it is not clear how six month follow up data could be analyzed among participants who receive intervention in the latter part of the six-month intervention implementation period. Time allocated for endpoint analysis is very brief.

Protections for Human Subjects:

Unacceptable Risks and/or Inadequate Protections

- "The T-Cup 16 panel Compact Instant Drug Test Cup with Alcohol provides results instantly, making it easy to use." Will test results, therefore, be communicated to study participants? How will referrals and linkage to services for help with reducing alcohol and drug use be handled, in cases of positive test results? Section states "all participants will be provided with information on alcohol and drug use, mental health, and other psychosocial support services that are available to them in their communities" but these resources are not named and procedures for linkage not described.

Data and Safety Monitoring Plan (Applicable for Clinical Trials Only):

Inclusion Plans:

- Sex/Gender: Distribution justified scientifically

- Race/Ethnicity: Distribution justified scientifically
- For NIH-Defined Phase III trials, Plans for valid design and analysis: Scientifically unacceptable
- Inclusion/Exclusion Based on Age: Distribution justified scientifically
- Measurements as described in research strategy are misaligned with outcomes described in statistical analysis plan section.

Vertebrate Animals:
Not Applicable (No Vertebrate Animals)

Biohazards:
Not Applicable (No Biohazards)

Applications from Foreign Organizations:
Justified

Resource Sharing Plans:
Acceptable

Budget and Period of Support:
Recommend as Requested

CRITIQUE 2

Significance: 3
Investigator(s): 2
Innovation: 1
Approach: 4
Environment: 1

Overall Impact: Applicants propose to assess alcohol and drug use (ADU) as well as contextually relevant risk and resilience factors in a sample of adolescents and youth living with HIV (AYLHIV) in a region of Uganda of predominantly of fishing villages where HIV is prevalent. Mixed methods including surveys to collect quantitative data and focus group discussions and key informant interviews will collect qualitative data. They propose to assess feasibility and short-term impact of a family-based economic empowerment (FEE) intervention on reducing ADU in a sample of AYLHIV in these communities which are highly impacted by poverty. The proposal is strong in its focus on improving understanding of multi-level context specific risk and protective factors for ADU among AYLHIV. There are many gaps in knowledge regarding ADU risk among AYLHIV in SSA. The investigative team is composed of a highly complementary group of researchers including early career and experienced mentors. The proposed work will address the stated aims and is not overly ambitious. However, the compression of the timeline in year two is worrisome. Some details are lacking in the Approach that would boost confidence in the ability to implement the study. If successful, the project will contribute

epidemiological data and contextual data regarding ADU in AYLHIV. The proposed study has potential to contribute important data and outcome knowledge to the field, potentially other interventions for AUD among youth and young adults, exerting medium impact due to some weaknesses in the approach.

1. Significance:

Strengths

- Alcohol and drug use (ADU) among adolescents and young adults in SSA contributes to HIV risk and among those living with HIV, poorer treatment outcomes. This study will contribute to filling gaps in knowledge regarding epidemiology, risk, and resilience factors among AYLHIV.
- AYLHIV in SSA experience significant stress, mental health challenges as well as negative social and structural challenges which increased risk for alcohol and drug use.
- The proposed study will be conducted in fishing communities located in three districts of the greater Masaka region of southwestern Uganda - a region heavily affected by HIV and high rates of household poverty and ADU.
- FEE intervention is novel, and the study proposes an appropriate pilot of this in a highly impacted population.

Weaknesses

- Little is stated about the effects – positive or negative of other economic-based interventions in this region or this population. What is the rationale behind thinking this will be equivalent or superior to others?

2. Investigator(s):

Strengths

- The MPIs bring highly complementary expertise to this study. Both are early career investigators with the background and experience to conduct this study.
- They have assembled an excellent team of investigators and collaborators, including senior researchers in the US and Uganda for the project.
- Strong partnership with Ugandan researchers.

Weaknesses

- An advising biostatistician would strengthen the study team.

3. Innovation:

Strengths

- Focusing on adolescents and young adults is not novel but is not common and thus is a strength. This is an ‘upstream’ looking proposal.
- FEE is a novel intervention for this target population.
- Biological measures including of ADU and viral suppression add value to the novel intervention.

Weaknesses

- None noted by reviewer

4. Approach:

Strengths

- The local implementation partner "Reach-the-Youth" has extensive experience and demonstrated experience with HIV-related trials.
- Embedding data collection with medical visits, for example urine screens could minimize refusal.
- RCT outcome measures are well described and feasible to collect.
- Methods for qualitative data collection and analysis are well described.
- Strong dissemination plan

Weaknesses

- Can the cross-sectional survey be administered without identifiers?
- How will the blinding of eligibility criteria be operationalized?
- Are investigators considering the survey to be the 'baseline assessment' for the trial? Or will they do a new assessment? If it is the baseline assessment there is a projected 4 months from end of survey to trial beginning, which could introduce error.
- Investigators do not include any feasibility measures for the pilot RCT (a stated goal in Aim 2)
- The primary components of the four financial literacy training sessions are not described. This seems a major omission as is it a key component of the intervention.
- Investigators do not provide estimates of confidence limits for point estimates (primary outcome) in the fixed sample size of n=200.
- Is there some assumption regarding the expectation that 50% (100/200) AYLHIV will be ADU positive?

5. Environment:

Strengths

- The researcher environment is excellent in Uganda and there is clear evidence of research strength there.
- The research environment sat WUSTL and Univ. of Michigan are outstanding.

Weaknesses

- None noted by reviewer

Study Timeline:

Strengths

- The major milestones required for this study to be implemented are mostly included. This includes primary goals for the qualitative and quantitative methods. However, it mostly focuses on processes and there is a need for documentation that any trial requires to be developed.

Weaknesses

- Suggesting having milestones for study materials including: standard operating procedures, manual of operations, Final Protocol, and Statistical Analysis plan

- The most intensive part of the project – developing and implementing the intervention is very compressed. Protocol development and finalization, which I believe will occur during the period labeled “intervention refinement and prep’ is targeted for 6 months, leaving very little room for any problem solving that occurs during the intervention implementation. Also, the FGD that take place following the intervention is compressed into 1 month. I would suggest trying to compress more into earlier time frame to have wiggle room for implementation and analytic challenges at the end.

Protections for Human Subjects:

Acceptable Risks and/or Adequate Protections

- US Certificate of confidentiality does not protect foreign participants. Are there potential safe
- Investigators need to have a plan regarding if they can and if so, how they will keep results of ADU testing confidential (from parents and providers). They state in the application that they will not share data, but what if pressure is exerted? What is acceptable and legal in Uganda regarding information collected from minors?

Data and Safety Monitoring Plan (Applicable for Clinical Trials Only):

Unacceptable

- o A data safety and monitoring board should be convened. A priori stopping rules should be considered.

Inclusion Plans:

- Sex/Gender: Distribution justified scientifically
- Race/Ethnicity: Distribution justified scientifically
- For NIH-Defined Phase III trials, Plans for valid design and analysis: Scientifically acceptable
- Inclusion/Exclusion Based on Age: Distribution justified scientifically

Vertebrate Animals:

Not Applicable (No Vertebrate Animals)

Biohazards:

Not Applicable (No Biohazards)

Applications from Foreign Organizations:

Justified

Resource Sharing Plans:

Not Applicable (No Relevant Resources)

Budget and Period of Support:

Recommend as Requested

CRITIQUE 3

Significance: 2
Investigator(s): 1
Innovation: 2
Approach: 3
Environment: 1

Overall Impact: This study aims to identify the multi-level (individual, interpersonal, community and structural) factors associated with AYLHIV and employ a family-based economic empowerment (FEE) intervention to prevent ADU among AYLHIV by reducing poverty and its associated mental impacts. It is of high public health impact given that in addition to inattention to AYLHIV, the limited number of prior AUD interventions that have been tested in South Africa have been largely ineffective potentially given focus only on individual and intra-personal risk factors. The MPIs have complementary expertise and history of collaboration and are supported by a strong team of co-Is with expertise in substance use, HIV, FEEs.

1. Significance:

Strengths

- Lack of evidence-based interventions targeting alcohol and drug use (ADU) risk among adolescents and youth living with HIV (AYLHIV) despite high prevalence of both among youth in fishing communities of Uganda.
- In addition to inattention to AYLHIV, the limited number of prior AUD interventions that have been tested in South Africa have been largely ineffective potentially given focus only on individual and intra-personal risk factors; this study will identify the multi-level (individual, interpersonal, community and structural) factors associated with AYLHIV and test an intervention that considers these.
- Intervention targeting this age group (15-24 years) is significant given that alcohol use nearly doubles during transition to adulthood from 21.4% of adolescents aged 16-19 to 34.1% of young adults aged 20-24 years.
- Builds off premise that family-based economic empowerment (FEE) interventions have the potential to prevent ADU among AYLHIV by reducing poverty and its associated mental impacts, and also bolster AYLHIV and their families' resources to overcome the challenges associated with HIV. Team has conducted previous studies and utilized FEE strategies to reduce poverty, improve mental health and HIV care outcomes among AYLHIV in Uganda.
- Aims to not only prevent but reduce AUD once started

Weaknesses

- Would have been useful to have information on the "value" of the actual amount of money to understand how this amount could reduce poverty in a meaningful and sustained way?

2. Investigator(s):

Strengths

- The MPIs have complementary expertise and history of collaboration and are supported by a strong team of co-Is with expertise in substance use, HIV, and experience with family-based economic empowerment interventions to address the HIV continuum
- MPIs have developed a strong working relationship as trainees of the LEAD Global Training Program and the Researcher Resiliency Training Program and have a shared mentor (Dr. Fred Ssewamala, co-I on the grant). MPIs are collaborating on several manuscripts and research proposals based on NIH-funded SUUBI studies.
- -Dr. Brathwaite is a postdoctoral research associate at WUSTL with training in Epidemiology and Population Health. She has research experience in substance use disorders and mental health among vulnerable/marginalized populations including intervention research and analysis focused on the effects of schools-based mental health interventions and the impact of family-based economic empowerment interventions on adherence, economic and educational outcomes among AYLHIV in low resource communities in Uganda. Dr. Brathwaite will lead the quantitative phase including quantitative data analyses for Aim 1b and Aim 2 of the study including analysis of data from the cross-sectional study and randomized controlled trial
- -Dr. Mutumba is an Assistant Professor at the University of Michigan with extensive experience conducting research among AYLHIV in Uganda. She has worked on several HIV clinical trials, managed complex national studies in SSA, and has extensive clinical and research experience working on adolescent substance use and mental health among AYLHIV across a range of settings. Dr. Mutumba will be responsible for the overall direction, implementation and administration of the study

Weaknesses

- None noted

3. Innovation:

Strengths

- Economic empowerment applied to AUD; includes role of poverty and families. Only one prior intervention has targeted the family
- Addresses the fact that ~89% of alcohol consumed in Uganda is homemade which makes it difficult to determine the quantity of alcohol consumed.

Weaknesses

- None noted

4. Approach:

Strengths

- Biologic measures to overcome challenges associated with self-reported AUD and address inconsistencies in self-report related to high prevalence of consumption of homemade alcohol and the normative nature of alcohol use in the community.
- Focus on multiple levels
- Recruitment strategies will build on Reach the Youth (RTY) (local implementing partner) and ICHAD's long-standing relationships (>15 years) with 39 health clinics in the greater Masaka region. All participants will be recruited from the six selected HIV clinics

- Youth Development Savings Account (YDA)- emphasizes long-term investment and promote life-long financial inclusion. Deposits made by the adolescent and family are matched by the intervention to encourage savings
- Randomization at the clinic level - inclusion of 6 clinics

Weaknesses

- Conceptual approach is informed by the socio-ecological model (SEM), social causation, drift theories and asset theory. While each model/theory independently seems appropriate and relevant to intervention, what is less clear is how these four works together and how their relative contribution to potential intervention efficacy will be tested.
- Are there any ethical considerations to not providing control group with money given exceedingly high rates of poverty? Any concern for potential contamination bias? What happens if they can't save anything? Is there any bare minimum that deposited?
- Among 200 participating in survey plan to enroll 100 (50%) who screen positive for AUD to participate in the randomized control trial and willing to participate. While rates of AUD high, based on data cited might 50% be too high?
- Eligibility based on screening positive at cross-sectional survey done at Months 10/11, while RCT not set to begin until Month 16. Urine test can detect alcohol from 8-80 hours after consumption and drugs from 1 hour to 40 days. Thus, likely that participants who were positive during survey will screen negative at baseline RCT assessment but will still be enrolled. How will this be handled in analysis?
- How assessing improved economic outcomes [secondary hypothesis]?

5. Environment:

Strengths

- Washington University and University of Michigan have resources to support MPIs in this endeavor
- Robust facilities at ICHAD (US and Uganda)
- Strong LOS from Uganda
- LOS from Equity Bank

Weaknesses

- None noted

Study Timeline:

Strengths

Weaknesses

- 2 months to enroll 200 AYLWH- No alternative plans for recruitment
- Among 200 participating in survey plan to enroll 100 (50%) who screen positive for AUD to participate in the randomized control trial and willing to participate- No alternative plans for recruitment [should recruitment or enrollment deviate we will schedule conference calls to enact solutions and adjust analytic strategy]

Protections for Human Subjects:

Acceptable Risks and/or Adequate Protections

- Are there any ethical considerations to not providing control group with money given exceedingly high rates of poverty?

Data and Safety Monitoring Plan (Applicable for Clinical Trials Only):

Acceptable

Inclusion Plans:

- Sex/Gender: Distribution justified scientifically
- Race/Ethnicity: Distribution justified scientifically
- For NIH-Defined Phase III trials, Plans for valid design and analysis: Scientifically acceptable
- Inclusion/Exclusion Based on Age: Distribution justified scientifically

Vertebrate Animals:

Not Applicable (No Vertebrate Animals)

Biohazards:

Not Applicable (No Biohazards)

Resource Sharing Plans:

Acceptable

Budget and Period of Support:

Recommend as Requested

THE FOLLOWING SECTIONS WERE PREPARED BY THE SCIENTIFIC REVIEW OFFICER TO SUMMARIZE THE OUTCOME OF DISCUSSIONS OF THE REVIEW COMMITTEE, OR REVIEWERS' WRITTEN CRITIQUES, ON THE FOLLOWING ISSUES:

PROTECTION OF HUMAN SUBJECTS: UNACCEPTABLE. See Reviewer 1 comments.

INCLUSION OF WOMEN PLAN: ACCEPTABLE

INCLUSION OF MINORITIES PLAN: ACCEPTABLE

INCLUSION ACROSS THE LIFESPAN: ACCEPTABLE

COMMITTEE BUDGET RECOMMENDATIONS: The budget was recommended as requested.

Footnotes for 1 R21 AA030225-01; PI Name: Brathwaite, Rachel

NIH has modified its policy regarding the receipt of resubmissions (amended applications).See Guide Notice NOT-OD-18-197 at https://grants.nih.gov/grants/guide/notice-files/NOT-OD-18-197.html. The impact/priority score is calculated after discussion of an application by averaging the overall scores (1-9) given by all voting reviewers on the committee and multiplying by 10. The criterion scores are submitted prior to the meeting by the individual reviewers assigned to an application, and are not discussed specifically at the review meeting or calculated into the overall impact score. Some applications also receive a percentile ranking. For details on the review process, see http://grants.nih.gov/grants/peer_review_process.htm#scoring.

MEETING ROSTER

The roster for this review meeting is displayed as an aggregated roster that includes reviewers from multiple CSR Special Emphasis Panels of the Risk, Prevention and Health Behavior Integrated Review Group
for the 2022/01 council round.
This roster for CSR is available at:
http://public.era.nih.gov/pubroster/Reports?DOCTYPE=SEP&DESFORMAT=PDF&AGENDA_SEQ_NUM_P=435514
